# A Pumpkin-Based Emulsion Gel as a Texture Improvement of Mixed Horsemeat Semi-Smoked Sausages

**DOI:** 10.3390/foods11233886

**Published:** 2022-12-01

**Authors:** Rysgul Ashakayeva, Bakhytkul Assenova, Galiya Tumenova, Almagul Nurgazezova, Gulnara Zhumanova, Zhibek Atambayeva, Assemgul Baikadamova, Dmitrii Il, Assel Dautova

**Affiliations:** 1 Department of Technology of Food Production and Biotechnology, Shakarim University of Semey, Semey 070000, Kazakhstan; 2 Department of Food Security, Manash Kozybayev North Kazakhstan University, Petrapavlovsk 150000, Kazakhstan

**Keywords:** horsemeat, pumpkin, quality of sausages, technological properties, emulsion gel, semi-smoked sausages, healthier meat products

## Abstract

Semi-smoked sausages were made with 5%, 10%, 15%, 20%, and 25% replacement of horsemeat by emulsion gel made with offal broth (stomach, kidney, liver, heart, brain, and a miscellaneous trimmings of a horse), pumpkin flour, and egg yolk in a ratio of 5:4:1. The technological, nutritional, oxidative, and rheological (G′ and G″) properties were studied. Sausage water holding capacity (WHC) rose after being incorporated with pumpkin-based emulsion gel (PEG). There was a statistically significant (*p* < 0.01) improvement in sausage emulsion stability. Lipid oxidation in all samples, especially 5% and 15% addition of emulsion gel samples, was below the rancidity criterion, which is TBARS > 2.0–2.5 mg MDA/kg sample. This really is encouraging because unsaturated fatty acids, such as those found in horsemeat, are easily oxidized. Use of the emulsion gel did not noticeably alter the sausages’ pH. Using emulsion gel considerably reduced the cooking loss (*p* < 0.05) of sausages and significantly improved texture (*p* < 0.05). Partial replacement of mixed horsemeat with emulsion gel improved the physicochemical characteristics of semi-smoked sausages. The elasticity modulus (G′) showed that PEG15 (15% of emulsion gel) was the most resilient gel. The least powerful gels (*p* < 0.05) were PEG20 and PEG25. According to this study, adding a pumpkin-based emulsion gel to the meat matrix could improve the quality of the emulsified meat system and provide important data for related research and companies as strategies to market a healthier and more nutritious product with the necessary quality characteristics.

## 1. Introduction

Due to their high protein content and improved nutritional value, meat and meat products constitute a substantial part of the human diet worldwide. As a result of its nutritional benefits—including a low intramuscular fat and cholesterol content and a high level of readily available iron-horsemeat has been promoted as a healthy addition to the human diet [1,2]. Fatty acids that are both monounsaturated and polyunsaturated can be found in high concentrations in horsemeat. In addition to being a rich source of minerals (particularly iron, phosphorus, zinc, magnesium, and copper) and essential and non-essential amino acids, it is also more easily digested than other red meats like lamb, beef, or pork [3,4,5]. Therefore, horsemeat is recommended in dietetic nutrition, as well as the nutrition of children, athletes, and people with anemia [3,5,6]. This rise in popularity coincides with the rise in interest in healthy eating. In order to meet the growing demand for horsemeat, attempts have been made to improve the horsemeat’s sustainability and safety. Emulsification technology could be used to improve the quality of semi-smoked sausages made from horsemeat by helping to increase their viscosity and stability, as well as control the moisture of the product [7].

Food items based on emulsions show a great deal of promise, both in terms of health and financial gain. Producing functional foods, which are foods that have been fortified with additional nutrients to increase their health benefits, is a potential new area of research. Soft, solid emulsion gels are preferable to classic emulsions because they can be used to create fat alternatives. In terms of texture, hardness, and water-retention capacity, among other features of animal fats, they are more authentic representations of animal fat [8,9]. The nutritional qualities of meat products can be enhanced by using these emulsions, which are better suited for transporting and safeguarding oxidized lipids in food and are more successful in maintaining taste components and bioactive chemicals. Improving our knowledge of how emulsion gels act in meat systems is crucial for ensuring high-quality end results. In addition, meat emulsions [10], patties [11,12], and sausages [13,14,15] might benefit from the use of emulsion gels rather than traditional oil-in-water emulsions due to the gels’ greater water-holding capacity, improved texture, and reduced cooking loss. Oxidation and quality loss can occur in meat products like horsemeat that are high in mono- or polyunsaturated acids. Therefore, extra components with high antioxidant activity would be helpful to prolong the process before oxidation of food sets in [16]. The gelled structure of an emulsion presents a chance to include oxidatively stable substances in meat products, hence extending their shelf life.

Because of their propensity to boost the meat’s health benefits and improve the product’s overall quality, fruits and vegetables are another source of interest in meat product formulations [17]. Bulambaeva et al. [18] showed that oxidation in sausages was decreased by adding pumpkin powder (*Cucurbita moschata*) and goji berries. There is evidence that pumpkin’s nutritional components can help fight diabetes, cancer, and weariness [19]. The high nutritious content of the pumpkin makes it a versatile vegetable (including carbs, vitamins A and C, carotenoids, and vital amino acids). It is an antioxidant [20] and is employed in the production of many manufactured goods. Since pumpkin pectin is a hydrocolloid, it can be used as a stabilizer in emulsions to improve texture, bind water, or thicken food [21]. According to research by Kim et al. [22], including just 2% pumpkin dietary fiber in low-fat frankfurters significantly altered their technological qualities. In their study of a 10% of pumpkin flour (PF)substitution, Hleap-Zapata et al. [23] discovered that the sausages’ water-holding capacity increased by 3.81%, while Unal et al. [24] informed that the adding of pumpkin powder enhanced the color, emulsion capacity (EC), emulsion stability (ES), and textural aspects of the emulsified meat products. These features suggest that adding pumpkin powder to meat products as an emulsion ingredient may improve their anti-oxidative and functional properties. As far as we can tell, there is no published research on the topic of incorporating pumpkin powder-based gel into a mixed horsemeat emulsion. In light of the preceding, the aim of the present study was to examine the effect of pumpkin powder-based emulsion gel on the qualitative aspects of semi-smoked sausage in an effort to broaden the range of horsemeat semi-smoked sausages currently available.

## 2. Materials and Methods

### 2.1. Materials

Fresh, non-damaged whole butternut squash (*Cucurbita moschata*) and spices were selected from the local marketplace in Semey and stored at 4 °C until used. The horsemeat (highest grade and grade I) and poultry (chicken) meat were supplied from local farms (“Nurbol” and “Klar” peasant farms, East Kazakhstan). The muscles were cleaned of any excess fat and connective tissues and kept at a 4 °C with fat until needed. All chemicals used in the research study were of analytical grade.

### 2.2. Pumpkin Flour Processing (PF)

**Figure 1:**
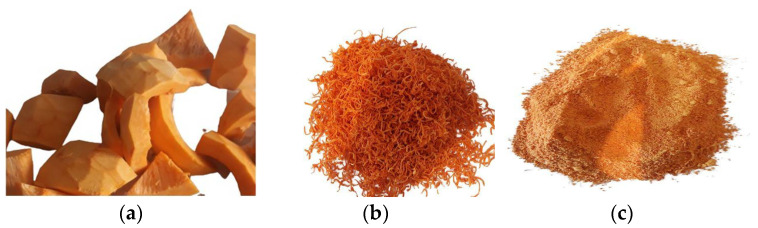
The process of the preparation of the PF ((**a**)—cutting process, (**b**)—after the dryer, (**c**)—pumpkin flour).

The PF was produced at Shakarim University of Semey (Semey, Kazakhstan) in the Department of Technology of Food Production and Biotechnology’s Laboratory of Operating System. At the Semey regional market, 8 kg of pumpkins (*Cucurbita moschata*) were purchased, all of the same ripeness. A sodium hypochloride solution at 700 ppm at 10 ± 1 °C was used to wash and disinfect pumpkins. The next step was to manually peel them, which entailed removing the seeds and husks. After it had been cleaned, it was shredded manually into pieces no bigger than 3–4 mm thick using a sharp stainless steel knife. Pumpkin was dehydrated in a tray dryer at 40–60 °C for 5 h (Sedona SD-9000, Tribest, Seoul, South Korea). After drying, the pumpkin was processed in a household grinder (Magic Bullet, Capital Brands, Los Angeles, CA, USA) until 95% of the particles went through a 12-mesh filter, or a particle sizes of 102 microns. The flour was stored in a dry, dark room at 24 ± 2 °C after being vacuum-packed in polyethylene bags using a vacuum baler (Boxer 42, Henkelman, Hertogenbosch, The Netherlands). In order to prevent light from interacting with the PF and changing its physicochemical, color, and textural properties, the bags containing the PF were wrapped with aluminum sheets. The content of mass fractions in 100 g of flour was as follows moisture 6.8%, proteins 11.51%, lipids 6.90%, carbohydrates 51.15%, minerals 15.40%, β-carotene 67.50 mg, and vitamin C 83.90 mg. The process of the preparation of PF is given in Figure 1. The method makes it possible to obtain a finished product powder from pumpkin pulp, which remains as a result of the industrial processing of pumpkin in the form of a powder with nutritious and beneficial properties. With these numbers, we were able to recreate 0.47 kg of PF.

### 2.3. Preparation of Pumpkin Based Emulsion Gel (PEG)

The preparation of the emulsion gel occurs in the cutter with the following feed loading scheme. To prepare 100 kg of PEG, into 50 kg of bouillon from cooking offal (35–40 °C) in the cutter with a knife speed of at least 3000 rpm, and a bowl of 12 rpm, added 40 kg of PF, mixed for 3–4 min, then added 10 kg of egg yolk and mixed for 4–6 min until a homogeneous emulsion is obtained. After this process, the mixture is unloaded into trolleys with a layer thickness of not more than 40 cm and directed for cooling to a temperature in the center of the product of 4 ± 1 °C. For this research, the emulsion gel (EG) sample was developed in a ratio of Broth: Pumpkin Flour: Egg Yolk as 5:4:1. This ratio was based on the findings of a preliminary study of functional properties such as gelling capacity, emulsifying activity, particle size of pumpkin powder, and texture of the final product. The formulation of PEG was selected after several trials. The pH value of the PEG was recorded as 6.37.

### 2.4. Analysis of the PEG

A pH meter (ST-2100; Mettler-Toledo, Greifensee, Switzerland) was used to take readings from multiple locations within the sample using a penetrating probe to determine the pH. The CIELab color system was used to evaluate the L* (lightness), a* (redness), and b* (yellowness) parameters of the instrumental color with a Konica Minolta CM-5 (Konica Minolta, Chiyoda-ku, Japan) spectrophotometer (D65 illuminant, 100 observer angle, and SCE mode). At 25 °C, each sample was analyzed three times.

A controlled-stress rheometer AR 1500ex (CPI, Liverpool, UK) with a 4 cm diameter stainless steel roughed parallel plate geometry was used to conduct the oscillatory rheological testing (gap of 1.5 mm). Prior to measurements, the samples were balanced for 2 min at 4 °C after being carefully placed on the plate using a laboratory spatula. 10 Pa of pressure was applied, at a frequency ranging from 1 to 10 Hz. Triplication was used to finish the analysis.

### 2.5. Preparation of Sausages with PEG

The formulation of the “Narli” semi-smoked sausages was developed in the Department of Technology of Food Production and Biotechnology of the Shakarim University of Semey. The formulations of “Narli” semi-smoked sausages with different additions of PEG are given in Table 1. The sausages were made with a mixture of horsemeat (highest grade and grade I) and poultry meat without any connective or adipose tissue. The horsemeat (highest grade and grade I) and poultry meat are crushed on a top with a lattice hole diameter of 8–12 mm. Shredded raw meat is subjected to a salting process by keeping it in a salt solution at 0–4 °C for 6–12 h. After the salting process, the PEG, water (ice), and potato starch are added. Next, the spices were added. The batter inside the cutter did not go above 12 °C. Within a maximum of 10 h, a sample of the batter vacuum-sealed into plastic bags (30 cm × 50 cm) and stored at 4 °C, was sent for subsequent assessments of its pH, color, rheological, and emulsion stability. Fibrous casings (Ø 45 mm, 600 g of product per unit) were used for the other part of the formulation. The formed loaves are subjected to curing for 2–4 h at 10–12 °C. After the curing process, the sausages proceed to roast at 60–90 °C for 30–60 min. Roasted loaves are steamed in steam chambers or boilers at 60–75 °C for 35–60 min. Boiling is completed and held up to the internal temperature of the sausages got 70–72 °C. Afterwards, the sausages are chilled to a temperature not exceeding 12 °C for 3–4 h. The cooling with water under a shower lasts 10–15 min, while the temperature inside the loaf drops to 30–35 °C. Next, smoking is carried out at 50 °C for 12–24 h and drying at 12–15 °C with air exchange for 2–4 days. Upon passing quality assurance, items are packaged, labeled, and held at around 5 °C for up to five days before undergoing pH, color, texture, and microstructural studies. For this research, five samples were developed with the content of PEG of 5%, 10%, 15%, 20%, and 25%, with a control sample without any PEG addition.

### 2.6. Analysis of Meat Batters Prior to Thermal Processing

#### 2.6.1. Rheological Properties

Frequency and stress (0.1–100 Hz) sweeps were completed as prescribed in Section 2.4. The formulation’s contents can cause variation in meat batters, even when other processing conditions, such as knife speed, temperature, and cutting time, are held constant. So, following the salting process, the combined mince paste was blended, and before the addition of the other components, 20 g of mince paste was taken and held at 4 °C overnight in order to analyze the actual rheological behavior of combined meat with emulsion gel. Raw mixed horsemeat emulsions were examined for their dynamic viscoelastic behavior using the procedure outlined in [25]. Specifically, 3 g of the uniform batter samples were put onto the rheometer’s bottom plate, left at room temperature (25 °C) for 5 min, and then subjected to a thermal sol-gel transformation ramping from 25 to 90 °C at a constant rate of 1 °C/min. The environment’s temperature was meticulously monitored and adjusted. A layer of silicon oil was applied to the exposed sample periphery to prevent dehydration. The gelation tests were conducted at a constant frequency of 1 Hz and a controlled stress of 50 Pa, both of which were chosen to correspond to the linear viscoelasticity range (LVR, 0.1–1000 Pa and 0.1–100 Hz, respectively). Both the storage modulus G′ (a measure of elastic property) and the loss modulus G″ (a measure of viscous property) were used to describe the outcomes.

#### 2.6.2. Emulsion Stability (ES)

ES was measured in accordance with [26]. In order to test the batter’s consistency, 25 g was weighed out and centrifuged at 2600× *g* for 5 min in 50 mL graduated tubes. The samples were heated in a water bath until the temperature in the center got 75 °C (25 min at 40 °C and 5 min at 75 °C). The tubes were then cooled to room temperature (25 °C), and the total fluid released was reported as a percentage of the sample’s weight. The treatment was performed in triplicate.

#### 2.6.3. The Color and pH

The color and pH of the batter were measured as demonstrated in Section 2.4. The batters were stacked into the cuvette to a height of about 1 cm for the color measurements. All measurements were taken at 25 °C in triplicate. In order to evaluate the differences between the control sample (C) and the PEG-added products (PEG), the Chroma value ($Chroma=\sqrt{a^{*2\;}+b^{*2}}$) and Hue angle ($Hue=\tan^{-1}\frac{b*}{a^{*}}$) were computed.

### 2.7. Evaluation of “Narli” Semi-Smoked Sausage

#### 2.7.1. Chemical Composition and Energy Value

Chemical composition and energy value (moisture, protein, ash, and fat content) were determined according to the requirements of GOST (State Standard) R 51479-99 (oven drying method), GOST 25011-81 using the Kjeldahl method, GOST 31727-2012 (muffle furnace), and GOST 23042-86 using the Soxhlet method. Analyses were completed three times. The energy value was designed based on the caloric content of fat (4.6 kcal/g), protein (21.42 kcal/g), and carbohydrate (0.077 kcal/g).

#### 2.7.2. The Color and pH Examination of “Narli” Semi-Smoked Sausage

The color and pH examination were done according to Section 2.4. The sausage was cut up to a depth of 0.5 cm to examine the color characteristics. The analyses were completed at 25 °C in triplicates. Hue and Chroma values were calculated as shown in Section 2.6.3.

#### 2.7.3. Texture Profile Analysis (TPA)

All texture readings were taken with a TA-XT Plus at a temperature of 4 °C (Stable Micro Systems, England). The sausages’ texture profiles were analyzed using a P-35 probe and the procedure outlined in [27]. The samples were acquired in the form of twelve metallic cylinders with dimensions of h = 20 mm and d = 20 mm. The samples were compressed in a single direction at 30% of their original height at a continual rate of 1 mm/s for two cycles. Hardness (N), springiness (mm), cohesiveness, chewiness (N*mm), and gumminess (N) were the measured properties.

#### 2.7.4. The Fatty Acid Profile

The product’s fatty acid profile was calculated by isolating lipids from samples by chloroform/methanol extraction, as described by the Folch method. Thin-layer chromatography was utilized to examine the extracted lipids for purity. Fatty acid composition was measured using an Agilent Technologies, USA HP-Innowax 30 m × 0.25 mm × 0.25 m column on a Hewlett Packard HP 6890 gas chromatograph with a flame ionization detector. The analysis was performed in triplicate.

#### 2.7.5. Lipid Oxidation

The extraction method was used to assess the TBARS (thiobarbituric acid reactive compounds) concentrations in four separate samples [28]. The absorbance was determined at 532 nm using a spectrophotometer (Beckman, DU-70, Triad Scientific, Inc. Manasquan, NJ, USA) and a blank solution of 3 mL trichloroacetic acid and 5 mL thiobarbituric acid (TBA) reagent. The outcomes were converted to milligrams of malondialdehyde per kilogram of meat (mg MDA/kg) using a TEP standard curve (1,1,3,3-tetraethoxypropane).

#### 2.7.6. Water-Holding Capacity (WHC) and Cooking Loss (CL)

WHC of sausages was analyzed according to [14]. CL was identified through calculation of the ratio of meat batter before and after cooking, and stated as follows:

$$\%\;\mathrm{Cooking}\;\mathrm{Loss}=\left(\frac{{\mathrm{weight}}_{\mathrm{raw}}-{\mathrm{weight}}_{\mathrm{cooked}}\;}{{\mathrm{weight}}_{\mathrm{raw}}}\right)\times 100$$

#### 2.7.7. Scanning Electron Microscopy (SEM)

The surface morphology of the sausage was assessed using a non-destructive technique known dispersing preparation of the sample using a high vacuum scanning electron microscope (TM 3000 Tabletop Microscope, Hitachi High Technologies, Tokyo, Japan), with a magnitude of 15× to 30,000× and a 15 kV acceleration voltage. We employed 100× and 200× magnifications. The product was put in a stub, cut into standard pieces (2 cm × 2 cm), and then evaluated at 15 kV in the modular apparatus.

### 2.8. Statistical Analyses

The testing was directed in duplicates. One-way analysis of variance (ANOVA) was used to analyze the data for the PEG (color, pH, emulsion stability), meat batter (color, pH, emulsion stability), and “Narli” semi-smoked sausage (proximate composition, pH, color, TPA, and TBARS), and Tukey’s test was used at 5% impact level (*p* < 0.05).

## 3. Results and Discussion

### 3.1. Property of Pumpkin-Based Emulsion Gel

The value of L* did not show a significant difference among PEG and horse fat samples (*p* > 0.05) (Table S1). Fat analog is darker than horsemeat fat, the main ingredient in making horsemeat sausages. Fat contributes to the meat’s tenderness and, in turn, the sensation of juiciness. Fat turns yellow and even orange on older animals [5]. Foals slaughtered at 9 months had a larger level of intramuscular fat than foals slaughtered at 12 months (*p* < 0.05), according to research by Franco et al. [29]. Therefore, similar outcomes necessitate considering the ages of the animals involved. The emulsion gel has a higher redness (a* = 1.36) and yellowness (b* = 7.90) value than the horse fat (*p* < 0.05). This effect may be due to the high-density yellow color of PEG itself. The pumpkin flour probably was responsible for these results due to high level of carotenoids, particularly, α- and β-carotene, β-criptoxanthin, lutein, and zeaxanthin. The flour dried at 45 °C preserved 95% of the α-carotene and 83% of the β-carotene [30]. Emulsion gel and horse fat both showed pH values near to neutral, therefore there wasn’t much of a difference between them (*p* > 0.05). Choe et al. [14] and Hleap-Zapata et al. [23], reported similar outcomes when utilizing a combination of pork skin and wheat fiber as a fat replacement and the partial replacement of wheat flour with different amount of pumpkin flour during development of frankfurt-type sausages. Frequency sweeps were used to acquire dynamic rheological measures. According to the values of the storage or elastic modulus (G′) and the loss or viscous (G″) modulus of the emulsion gel and control (HF) (not shown), the gel exhibited dense properties. Emulsion gels, on the other hand, showed greater G′ and G″ values than horse fat. Complexes of pumpkin flour and egg yolk formed at pH levels above the isoelectric point (5.7) of egg yolk. Previous findings in protein dispersions demonstrated the importance of electrostatic repulsions when the protein’s net charge increased as pH deviated from its isoelectric point (IEP), where the net surface charge was zero. Extreme pH levels cause protein unfolding, which in turn promotes protein aggregation and the establishment of a network [31]. Although egg yolk is most commonly employed to stabilize food emulsions, it also has the ability to gel, texture, and bind in baked goods. Egg yolk’s ability to gel is one of its most useful features. Gels can be formed from egg yolk because of its high protein content and wide range of lipids. Gel formation is due to interactions between proteins and between proteins and lipids, which occur when the natural structure of the proteins in egg yolk is disrupted by treatments like heat, alkali, salt, etc. An increase in G′ and G″ can be attributed to protein aggregation, which is in turn explained by partial protein denaturation, as shown by Clark et al. [32]. Finally, protein aggregates associate to create a three-dimensional network. The protein gel network may also be strengthened by some covalent crosslinking linkages [33]. Functional components, such as egg yolk and pumpkin flour, impact the emulsion gel’s stability. Since pumpkin flour is hydrophilic, colloidal, and water-holding, it improves the emulsion’s stability when added. According to Nidhal et al. [21], pumpkin flour works well as a stabilizer in low-calorie mayonnaise. Concentration of pumpkin powder and egg yolk proteins, temperature, pH, and ionic strength of the medium all play a role in the gel development qualities, net configuration, and rheological characteristics of the finished product [16].

### 3.2. Characteristics of Emulsion Gel-Made Sausage

#### 3.2.1. Proximate Composition

Table 2 displays the proximal compositional differences between semi-smoked sausages made with different PEG levels. The moisture content was higher in semi-smoked sausages with increasing PEG addition (*p* < 0.05) than that in the control sample, which could be attributed to the existence of water in emulsion gel along with preferred water binding ability contributed by the added pumpkin flour and due to greater protein and dietary fiber content of flour that led more water being entrapped in the meat matrix. Similar tendencies in moisture content were documented by Kim et al. [22] when varying amounts of fat and pumpkin fiber extract were added to reduced-fat frankfurters and Alves et al. [34] found a rise in the moisture content of sausages made using green banana flour gels as fat replacers. Moisture content is a significant property of sausages, because of its effect on the end product’s texture, sensory features, and ultimately its weight, all of which contribute to the products economic worth [15,35]. Fat contents in different sausages ranged from 22.28% to 7.35% (*p* < 0.05) and protein content ranged from 22.14% to 18.78% (*p* < 0.01) compared to the control sample. This finding is consistent with that of Ali et al. [36], who found that adding 10% rice flour to pig and duck sausage and Cittadini et al. [11], who reported that the replacement of 100% of pork fat with the oil mixture emulsions added avocado or pumpkin seed into foal burgers reduced the protein and fat content. Similarly, Öztürk-Kerimoğlu et al. [37] also found that the inclusion of quinoa flour improved moisture and carbohydrate contents however decreasing fat and energy values. So, the addition of quinoa flour successfully increased both protein and fiber content. On the other hand, the addition of PEG did not had significant affect on to the ash content. Semi-smoked sausages made with varied concentrations of emulsion gel had drastically variable calorie count (Table 2). The energy value tended to decrease by adding emulsion gel (*p* < 0.05). Substitution of beef fat with quinoa flour in low-fat sausages had comparable effects, as described by the authors [37]. According to Turhan et al. [38], total fat levels influenced the energy content of beef products because lipids supply 9 kcal/g of energy, which is much more than the energy given by proteins (4.02 kcal/g) or digested carbs (3.87 kcal/g). Similar outcomes were observed by Pintado et al. [16] when they used a chia and oat emulsion gels, while Bozhko et al. [39] used a soybean isolate to achieve the same effect to replace some of the pork.

#### 3.2.2. Fatty Acid Profile (FAP)

Table 3 shows that the addition of emulsion gel did not significantly alter the FAP of semi-smoked sausages, presumably because of the high content of unsaturated amino acids in horsemeat. Specifically, palmitic (31.2%), stearic (4.45%), and myristic (4.54%) fatty acids and oleic (34.21%), linoleic (16.6%), and -linolenic (4.1%) fatty acids made up the FAP of the semi-smoked sausage of the sample without PEG (control).

The analysis of the fatty acid content of mixed horsemeat semi-smoked sausage with PEG confirms that the concentration of total SFA in the formulations was reduced com-pared to control sample. Da Silva et al. [15] reported a significant reduction in SFA content by the substitution of up to 50% of pork back fat by oleogel. As compared with the control sample, the reduction of SFA in the PEG15 was greatest (13.71%) among the other treatments. The PEG did not had the noticeable affect on MUFA content of the semi-smoked sausages, probably due to the higher amount of MUFA in horsemeat (40.1%) compare to beef or pork [40]. Horsemeat is low in fat and has a high concentration of healthy omega-3 polyunsaturated fatty acids (PUFA), including linolenic (18:3n-3) and other long-chain n-3 fatty acids (FA), which have been shown the valuable effect to prevent some chronic diseases [1,3,4,6,7]. The ω-6/ω-3 ratio describes how healthier is meat products. Greater values of the ω-6/ω-3 ratio are connected with the growth of several disorders, including cancer and heart disease; the ideal ratio is <4 [41]. Our value is slightly higher because the formulation included horsemeat which is rich in PUFA. Our findings are consistent with prior research [39] showing that semi-smoked sausages made with duck meat have a significant polyunsaturated fatty acid content. Authors [42] also obtained a ratio for traditional sausages (8.26) over goods that were either locally sourced (16.80) or were conventional (13.75). A mean ratio of 13.87 was found by Pietrzak-Fieko and Modzelewska-Kapitua [43], whereas Amaral et al. [44] found a range of 9 to13 for pork frankfurter style sausages.

#### 3.2.3. The Color, pH, Emulsion Stability (ES), WHC, CL, and TBARS

Table 4 displays the results for pH, L*, a*, b*, Chroma and Hue values, and TBARS for semi-smoked sausage batter and sausages with varying levels of PEG.

Adding PEG had a slight tendency to raise the pH of the mixed meat batter and sausage, but there were not significant (*p* > 0.05) changes in the pH of the mixed meat emulsions that were formed using PEG. Similar findings were found by authors [22], when pumpkin fiber was added to lower the fat content of frankfurters, while studies by Serdaroğlu et al. [45] and Unal et al. [24] found that adding dry pumpkin pulp and seed combination to beef patties or pumpkin powder to beef emulsions raised the pH. To the contrary, Ahmed et al. [46] discovered that adding pumpkin powder to beef sausages caused the pH value to drop relative to the control samples. Water-binding capacity and emulsion stability are enhanced in meat systems with a high pH [47].

Consumers are heavily influenced by a product’s visual appeal, making color a crucial role in the evolution of meat products [48]. Color values for batters and semi-smoked sausages are displayed in Table 3 as L*, a*, b*, Chroma, and Hue angle. Since the PEG had a dark tendency that is very similar to that of the meat emulsion, it is clear that the addition of PEG did not result in significantly different L* values from the control sample (*p* > 0.05). L* values of beef patties containing pumpkin pulp and seed and beef burgers including pineapple, passion fruit, or mango byproducts were not significantly different, as was the case with Serdaroğlu et al. [45] and Selani et al. [49]. Contrarily, Kim et al. [22] and Öztürk-Kerimoğlu et al. [37] reported decreasing L* value in frankfurters by reducing the fat content level with added pumpkin fiber and in a combination of teff flour with quinoa flour in beef sausages. Freshly prepared sausage’s a* values decreased and b* values did not have a significant difference in comparison to the control sample (*p* > 0.05). Da Silva et al. [15] reported lower a* and higher L* and b* values of freshly prepared sausages with oleogel, whereas Calvalho Barros et al. [12] noted no change in a* value in beef burgers with tiger nut oil emulsion. The addition of pumpkin powder into a beef emulsion both boosted the emulsions’ yellowness (b*), as reported by Unal et al. [24]. Using amorphous cellulose fiber in place of pig fat has not been shown to appreciably alter the color characteristics, save for the change in b* values after storage, as previously reported by Schmiele et al. [50]. Based on these results, it appears that formulation components used in meat products may have varying effects on the end product’s color.

Dissimilarities in the light scattering properties of the meat fat and PEG in the sausage batters may be to reason for the differences in color factors among the control and PEG-added sausages [50], and the presence of polyphenolic compounds in pumpkin powder may be transferred to the final product, as shown by the Chroma values (Table 3), which indicates the color saturation. Similarly, the Hue angle, the indication of the presence of red, was at its minimum in the control sample and increased in intensity in the samples that included PEG [51].

Lipid oxidation is crucial to the storage, quality, and nutritional value of meat products. PEG5 and PEG15 showed lowest oxidation levels among all samples (Table 3). Since pump-kin is a source of carotenoids, tocopherols, and antioxidants it was expected that the in-corporation of PEG into sausage formulation will reduce the oxidation level. All samples had oxidation levels that were lower than the rancidity criterion, TBARS > 2.55–10.0 mg MDA/kg sample [52], with values below 0.2 mg MDA/kg sample. Our samples showed TBARS, (mg MDA/kg) values as Control (0.15 ± 0.03), PEG5 (0.06 ± 0.02), PEG10 (0.08 ± 0.02), PEG15 (0.06 ± 0.03), PEG20 (0.08 ± 0.01), and PEG25 (0.09 ± 0.02) mg MDA/kg, which are showing lower value than the rancidity level as per reference [52]. According to Zhang et al. [52] the meat remains acceptable to consumers even when their TBARS values achieve levels of 2.5 mg MDA/kg or 10.0 mg MDA/kg. Since unsaturated fatty acids found in horsemeat are easily oxidized, this is a positive finding. PEG’s ability to lower TBARS (*p* < 0.05) may be attributable to the antioxidant chemicals found in pumpkin in PEG, which slowed down the oxidation process. Wahyono et al. [53] observed similar results, stating that the addition of 20% hot air dried pumpkin powder reduced lipid oxidation because of the pumpkin’s natural antioxidants. Yet, a shelf-life investigation is required to verify the reduced lipid oxidation in semi-smoked sausages.

Emulsion stability (ES) and cooking loss (CL) are significant factors to be assessed for predicting the technological quality of sausages. Table 5 displays the ES and CL value of semi-smoked sausage with different amounts of PEG.

While all PEG-made sausages had lower CL than the control sample, PEG15 had the lowest CL (*p* < 0.05). Reformulated sausages using chia or oat emulsion gels as a fat replacer have been shown to be very resistant to cooking temperatures, as described by Pintado et al. [16]. Unal et al. [24] found a similar pattern, reporting that the addition of pumpkin powder to beef emulsions reduced CL and raised ES. The forms of dietary fiber added to the meat product have a significant impact on CL. Hence, the drop in CL might have resulted from the semi-smoked sausages’ enhanced water absorption capability due to the addition of dietary fiber in the form of PEG. The values of water and fat exudation ranged from 6.40–20.45 to 1.14–6.45, respectively (Table 5). The control sample showed higher water and fat exudation, and therefore, lower emulsion stability (*p* < 0.05). In addition, higher emulsion stability was observed with the increase in the replacement level (*p* < 0.05). These results can be due to the dietary fiber content of pumpkin in the emulsion gel, resulting in greater retention of water and lipids in the food matrix. Egg yolk and pumpkin powder serve to increase the emulsion’s stability. Pumpkin flour is used as a stabilizer in low-fat mayonnaise due to its hydrophilic colloidal qualities [21]. CL and ES values are in agreement with those published by Öztürk-Kerimoğlu [51], who found that elevating the concentration of the pea protein-agar agar gel complex enhanced the stability and CL. Similarly, Choe et al. [14] and Alves et al. [34] reported lower cooking loss and higher emulsion stability when a mixture of chicken skin and dietary fiber and pork skin and green banana flour were used as fat replacement in emulsified meat products. Protein source, fat amount, fiber type, salt concentration, and processing methods are all potential contributors to the ES of semi-smoked sausage batter, in addition to supplementary additives.

The presence of dietary fibre in pumpkin flour may be responsible for the rise in WHC. Water molecules fill the pore space of fiber particle as the dietary fibre hydrates. Additionally, the gelatinization of hydrated pumpkin flour at high temperatures may be the cause of the observed rise in WHC in the treated sample. Accordingly, Serdaroğlu et al. [45] reported that adding pumpkin mix to beef patties increased WHC. Ammar et al. [54] and Unal et al. [24] found that the incorporation of pumpkin powder into meatball samples increased WHC more than samples with date seed flour or wheat germ and physicochemical characteristics of beef emulsion improved by introducing pumpkin powder. Thus, for an acceptable cooking yield, a good water retention capacity is also required. To assure a juicy meat product after culinary treatment before consumption, it is crucial to combat the exudation inside the sausages’ package during storage and commercialization.

#### 3.2.4. Texture Analysis Profile

The texture of meat products is often improved by the addition of non-meat substances such soy protein, whey protein, and carbohydrates like starch and cereal flour. Table 6 provides information on the various sausage textures. The strong binding capacity of the fiber source components and egg protein likely contributed to the softer structure seen at higher concentrations of PEG (*p* < 0.05), which in turn reduced hardness. This suppleness might be a result of the higher moisture content as well. Similar reductions in hardness and gumminess were found by Öztürk-Kerimoğlu et al. [37,51] when they used a pea protein-agar agar gel complex and included quinoa flour and teff flour. Adding egg yolk protein to meat emulsions had a similar effect, increasing the hardness value while also increasing the capacity to absorb water [55]. Chewiness and gumminess scores for PEG-treated sausages were significantly lower compared to those for the control group. Considering the close relationship between softness and the other features, it is possible to implement these alterations in response to the reduced hardness value. The reduced hardness and chewiness of low-fat meat emulsions produced with pea protein-agar agar complex and aloe gels was also linked by Öztürk-Kerimoğlu et al. [51] and Kumar et al. [56] to increased water binding and fat capacity. Cohesiveness values (*p* < 0.05) that increase when PEG is added indicate that the PEG has been successfully incorporated into the meat mixture, which in turn suggests that the internal linkages in these samples are stronger than in others [56]. Both Öztürk-Kerimoğlu et al. [57] and Eim et al. [58] observed that the inclusion of carrot powder into beef meat batter and the addition of carrot dietary fiber to fermented sausages altered the hardness and gumminess of the final product, and our results corroborate their findings.

#### 3.2.5. Rheological Characteristics of the Meat Emulsion before Thermal Processing

**Figure 2:**
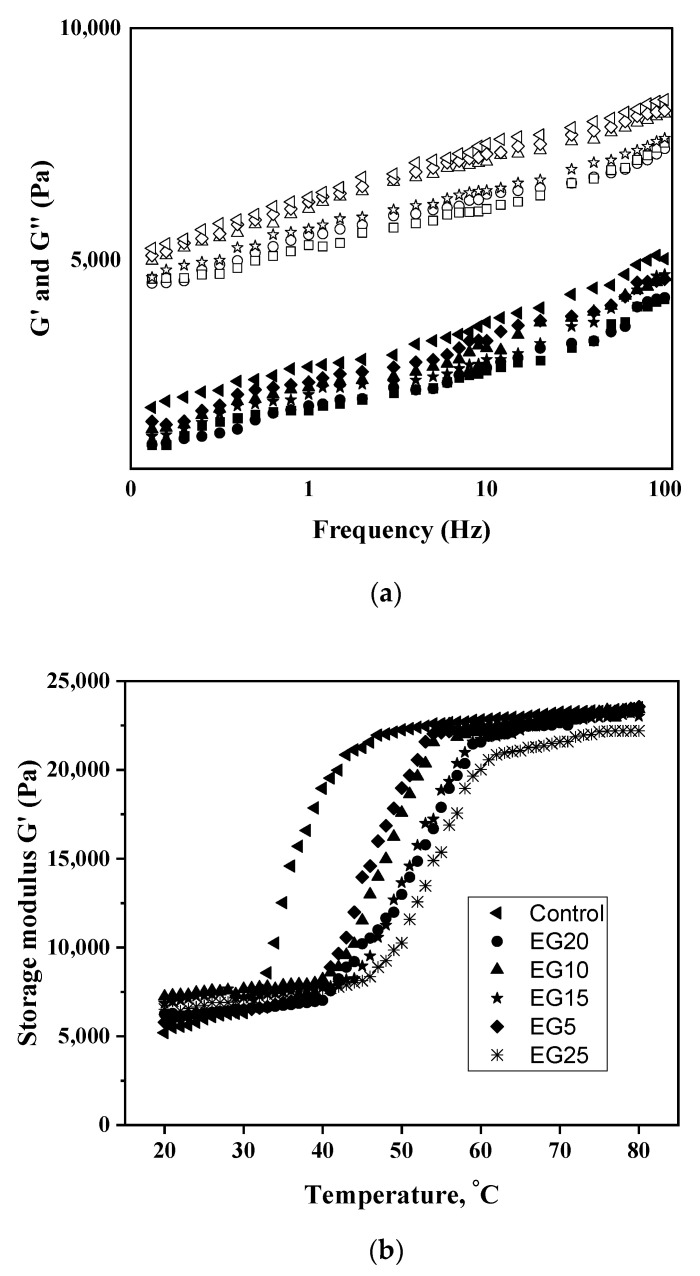

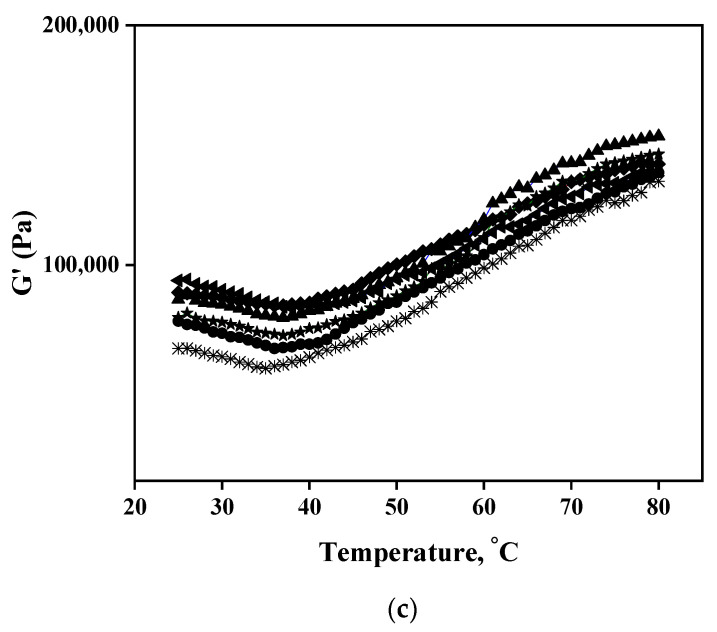
(**a**) Storage (G′, open symbols) and loss (G″, closed symbols) modulus of the meat batters as a function of frequency for Control (◀, ◁), PEG5 (◆, ◇), PEG10 (▲, △), PEG15 (★, ☆), PEG20 (●, ○), PEG25 (■, □); (**b**) Storage modulus (G′) of meat emulsions during heating; (**c**) and cooling for control (◀), PEG5 (◆), PEG10 (▲), PEG15 (★), PEG20 (●), PEG25 (✴).

The rheological measurements are very important to manage the chemical interactions of food components to get the desired food structure with the desired texture characteristics [59]. Figure 2 displays the storage (G′) and loss (G″) modulus of the meat batters (a). The paste exhibited characteristics of viscoelastic solids, with G′ values greater than G″ within the frequency range studied. All of the samples exhibited the characteristic viscoelastic behavior associated with ‘weak gel’ qualities, proving that the produced 3D gel cross-linked gel network was rheologically consistent across all of the samples [59]. Low-fat meat emulsions with fish oil and various binders [60] and low-fat sausage with inulin [61] as a fat substitute both had similar results. These characteristics are crucial when analyzing the potential uses of this emulsion gel in the meat processing business.

#### 3.2.6. The Influence of Heat on the Rheological Characteristics of the PEG-Added Meat Emulsion

During heating (25–80 °C), the structure of the semi-smoked sausages created with varying concentrations of emulsion gel are shown in Figure 2b as a function of storage modulus vs. temperature. Up to 80 °C, all treatments, with the exception of control, exhibited identical thermo-rheological curves. The three changes in G’ during heating suggest that protein denaturation occurred while the raw sausage was being heated. Heating the batter changes it from a disorganized system into a new well-ordered gel matrix (G′) with similar elastic properties across all treatments except at 45–80 °C [62], where covalent bonding and hydrophobic interactions for proteins are accomplished. When the temperature reaches this point, the viscous solution transforms into an elastic gel network, with G′ increasing as the temperature rises [62]. Compared to the other sausages, the control sample’s elastic network construction began earlier when no PEG was added. The dissimilarity amongst control and other formulations is probably associated with a more complicated configuration, necessitating more energy to breakdown the bonds and component connections that lead to gel formation. At temperatures around 50 °C, the myosin tail forms a semi-gel, which likely explains why the G′ peak rises slightly. After this stage, G′ decreased with increasing temperatures from 55 to 60 °C due to denaturation of myosin tails, which altered fluidity and disrupted the meat protein network established at lower temperatures. When the temperature was raised from 60 °C to 80 °C, the viscous sol transformed into an elastic gel network, and the G′ increased fast [62]. After the heating process was complete, G′ showed that all treatments were equally elastic and viscous.

During the cooling process, as depicted in Figure 2c, the G′ of meat emulsions continues to form and the components of the sausage partially crystallize, resulting in the rheological features of the final product [62]. The final readings of the elasticity modulus showed that PEG15 was the most resilient gel. The least powerful gels (*p* < 0.05) were PEG20 and PEG25.

#### 3.2.7. Microstructure (SEM Analyses)

**Figure 3:**
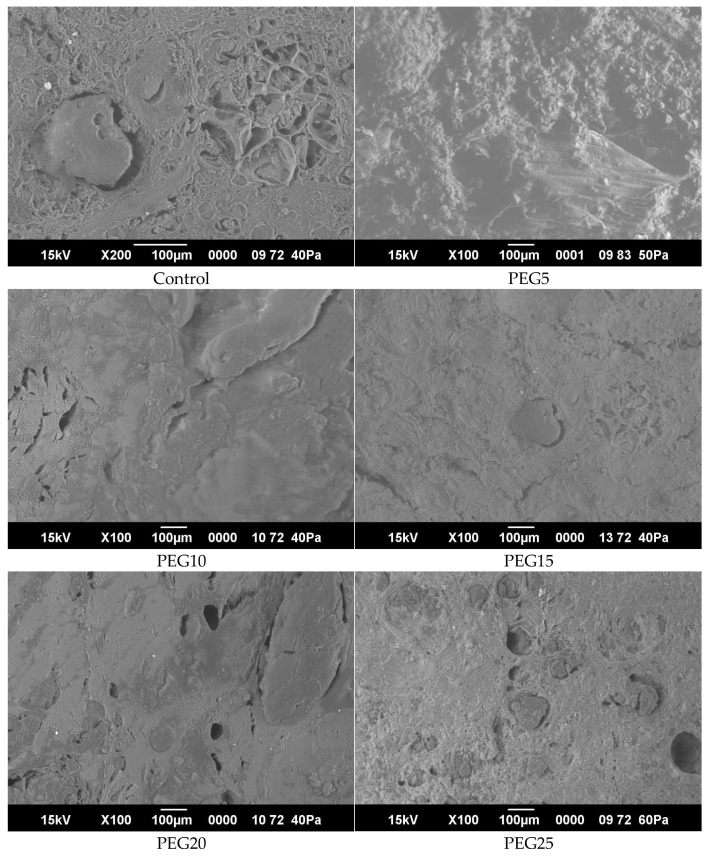
SEM images of semi-smoked sausages with different level of PEG.

The semi-smoked, mixed horsemeat sausages with emulsion gel can be seen in detail in the SEM images shown in Figure 3. There was no discernible visual difference between the untreated and treated samples, as shown by the SEM images. Water and air expansion caused the control sample’s irregular structure to seem spongy [63]. All the other samples had the same amounts of water (Table 1), but their structures were more structured and homogeneous. Pumpkin flour, which binds well to both water and fat, was used to create an emulsion gel for these formulations. When compared to other samples, the PEG15 exhibits the most homogeneous sausage matrix structure. These findings may be explained by the high antioxidant capacity of pumpkin flour (thanks to its high fiber and protein content and its position high in the β-carotine/linoleic acid system) [53], which allows having a good binding ability with water and fat. It was suggested that a chemical treatment to be performed on the meat sample prior to examination in order to separate the protein component, a fat globule, and a pore.

## 4. Conclusions

The incorporation of pumpkin-based emulsion in semi-smoked sausages made with mixture of horsemeat (highest grade and grade I) and chicken was analyzed. Sausage hardness, viscoelasticity, network structure, and cooking loss were all improved by the addition of an emulsion gel made from pumpkin powder, egg yolk, and bullion in the ratio of 5:4:1. Pumpkin powder, a rich source of antioxidants and fiber, can be used to make emulsion gel that can increase the sausages’ resistance to oxidation, which was noticed in samples PEG5 and PEG15. Yet, the study of lipid oxidation during storage is needed to understand the PEG property more clearly. Reformulation of the sausage presented greater effects on the physicochemical, rheological, and microstructural properties when emulsion was used in 15% substitution of the meat batter. Research from this study suggests that incorporating pumpkin flour into emulsion gel for use in the processing of meat products could be an effective method for improving the meat industry. In any case, the sensory analysis would be essential to the introduction of this new product. There will soon be a study comparing the effects of various antioxidant chemicals on the storage life of semi-smoked sausages.

## Figures and Tables

**Table 1:** The formulation for control and “Narli” semi-smoked sausages.

Ingredients	Control	PEG5	PEG10	PEG15	PEG20	PEG25
Horsemeat (high grade and grade I)	55	25 + 25	22.5 + 22.5	20 + 20	17.5 + 17.5	15 + 15
Poultry meat	30	30	30	30	30	30
Starch	5	5	5	5	5	5
Water (ice)	10	10	10	10	10	10
PEG	-	5	10	15	20	25
Total	100	100	100	100	100	100
Spices (per 100 kg of raw materials)						
Nitrite-salt mixture	1.7 kg	1.7 kg	1.7 kg	1.7 kg	1.7 kg	1.7 kg
Garlic	1 kg	1 kg	1 kg	1 kg	1 kg	1 kg
Nutmeg	100 g	100 g	100 g	100 g	100 g	100 g
Black pepper	300 g	300 g	300 g	300 g	300 g	300 g
Sugar	100 g	100 g	100 g	100 g	100 g	100 g

**Table 2:** Semi-smoked sausages’ proximate composition formulated with horsemeat and different level of emulsion gel.

Parameter	Control	PEG5	PEG10	PEG15	PEG20	PEG25	*p*-Value
Moisture (%)	66.37 ± 0.90 ^a^	68.18 ± 0.18 ^a^	71.08 ± 0.12 ^c^	71.83 ± 0.14 ^b^	71.08 ± 0.12 ^b^	72.12 ± 0.27 ^b^	*
Lipid (% u.b)	22.28 ± 0.67 ^a^	10.55 ± 0.81 ^c^	7.35 ± 1.00 ^a^	7.96 ± 0.30 ^bc^	8.52 ± 0.81 ^a^	9.97 ± 0.57 ^b^	*
Protein (% u.b)	22.14 ± 0.50 ^ab^	20.03 ± 0.36 ^ab^	21.50 ± 0.30 ^a^	19.72 ± 0.44 ^bc^	19.45 ± 0.01 ^a^	18.78 ± 0.01 ^c^	**
Ash (% u.b)	1.59 ± 0.10 ^a^	1.93 ± 0.04 ^a^	2.37 ± 0.05 ^a^	2.64 ± 0.07 ^a^	2.59 ± 0.06 ^a^	2.32 ± 0.09 ^a^	ns
Energy Value (Kcal/100 g)	226.17 ± 5.12 ^a^	212.52 ± 5.24 ^bc^	185.15 ± 4.21 ^c^	165.21 ± 4.39 ^a^	237.45 ± 4.84 ^c^	178.52 ± 5.21 ^a^	*

All values are mean ± standard deviation of triplicates. * There were no significant differences (*p* > 0.05) across samples with the same letter configuration in the same row. *p*-value: ** (*p* < 0.01), * (*p* < 0.05), ns (not significant).

**Table 3:** Semi-smoked sausages with varying emulsion gel concentrations (g/100 g product) in terms of fatty acid profile.

Fatty Acid (%)	Control	PEG5	PEG10	PEG15	PEG20	PEG25
Saturated fatty acids (SFA)						
C12:0 lauric	0.256	0.249	0.189	0.200	0.158	0.24
C14:0 myristic	4.54	4.54	3.52	4.35	4.20	4.15
C15:0 pentadecanoic	0.425	0.420	0.425	0.412	0.415	0.418
C16:0 palmitic	31.2	30.6	30.2	29.8	30.6	31.0
C17:0 margaric	0.485	0.479	0.385	0.363	0.411	0.423
C18:0 stearic	4.45	4.40	3.54	3.38	3.89	4.11
C20:0 arachidic	0.071	0.071	0.068	0.071	0.068	0.062
C22:0 behenic	0.108	0.091	0.105	0.108	0.085	0.095
Total SFA	41.535	40.85	38.432	35.842	39.827	40.498
Monounsaturated fatty acids (MUFA)						
C16:1, ω-7 palmitoleic	0.272	0.251	0.214	0.278	0.188	0.254
C17:1 heptadecene	0.13	0.13	0.13	0.13	0.13	0.12
C18:1, ω-9, cis oleic	34.21	34.11	33.85	34.28	34.11	34.15
C18:1, ω-9, trans oleic	0.117	0.108	0.113	0.116	0.114	0.114
Total MUFA	34.729	34.599	34.307	34.694	34.542	34.638
Polyunsaturated fatty acids (PUFA)						
C18:2 trans linoleic	0.12	0.11	0.12	0.12	0.105	0.10
C18:2, ω-6 cis linoleic	16.6	16.5	16.2	16.5	16.3	15.8
C18:3, ω-3 linolenic	4.10	4.13	4.10	4.18	3.52	3.64
C20:4, ω-6 arachidonic	1.68	1.65	1.58	1.68	1.25	1.53
Total PUFA	22.5	22.39	22.0	22.48	21.175	21.07
PUFA/SFA	0.542	0.547	0.570	0.625	0.532	0.520
ω-6/ω-3	4.46	4.43	4.44	4.23	4.98	4.76

**Table 4:** The color, pH, and TBARS parameters of mixed horsemeat emulsions and sausages.

Parameters	Control	PEG5	PEG10	PEG15	PEG20	PEG25	*p* Value
Meat batter							
pH	6.44 ± 0.07 ^b^	6.48 ± 0.12 ^ab^	6.45 ± 0.10 ^a^	6.46 ± 0.11 ^a^	6.57 ± 0.07 ^a^	6.56 ± 0.11 ^a^	*
L*	65.16 ± 0.13 ^a^	64.87 ± 0.91 ^a^	64.17 ± 0.88 ^a^	63.27 ± 0.56 ^a^	63.48 ± 0.16 ^a^	62.44 ± 0.78 ^a^	ns
a*	16.22 ± 2.51 ^a^	15.21 ± 1.15 ^a^	12.58 ± 1.43 ^c^	11.60 ± 0.69 ^b^	10.76 ± 0.59 ^b^	10.42 ± 0.87 ^b^	*
b*	11.45 ± 0.43 ^a^	11.61 ± 0.53 ^a^	11.89 ± 0.21 ^a^	11.77 ± 0.76 ^a^	11.69 ± 0.56 ^a^	10.97 ± 0.55 ^a^	ns
Chroma	19.85 ± 0.25 ^b^	19.13 ± 0.21 ^a^	17.31 ± 0.45 ^c^	16.53 ± 0.23 ^c^	15.89 ± 0.15 ^a^	15.13 ± 0.15 ^a^	**
Hue	0.61 ± 0.05 ^b^	0.65 ± 0.08 ^a^	0.75 ± 0.01 ^a^	0.79 ± 0.02 ^a^	0.83 ± 0.03 ^a^	0.81 ± 0.01 ^a^	*
Semi-smoked sausage							
pH	6.56 ± 0.13 ^b^	6.58 ± 0.09 ^ab^	6.61 ± 0.06 ^a^	6.67 ± 0.11 ^b^	6.73 ± 0.08 ^b^	6.76 ± 0.06 ^a^	*
L*	62.06 ± 0.67 ^a^	61.87 ± 0.91 ^a^	61.34 ± 0.81 ^a^	61.17 ± 0.91 ^a^	60.13 ± 0.61 ^a^	58.54 ± 0.78 ^a^	ns
a*	14.12 ± 2.51 ^a^	14.01 ± 1.11 ^a^	10.52 ± 1.31 ^b^	9.70 ± 0.97 ^c^	8.86 ± 0.86 ^c^	8.52 ± 0.92 ^b^	*
b*	12.36 ± 0.51 ^a^	12.41 ± 0.61 ^a^	12.76 ± 0.31 ^a^	12.57 ± 0.41 ^a^	12.69 ± 0.46 ^a^	12.67 ± 0.52 ^a^	ns
Chroma	18.77 ± 0.35 ^a^	18.72 ± 0.55 ^a^	16.54 ± 0.27 ^c^	15.88 ± 0.43 ^a^	15.48 ± 0.17 ^b^	15.26 ± 0.25 ^a^	*
Hue	0.72 ± 0.01 ^ab^	0.72 ± 0.05 ^a^	0.88 ± 0.02 ^ac^	0.91 ± 0.02 ^a^	0.96 ± 0.02 ^c^	0.97 ± 0.01 ^a^	**
TBARS, mg MDA/kg	0.15 ± 0.03 ^a^	0.06 ± 0.02 ^c^	0.08 ± 0.02 ^a^	0.06 ± 0.03 ^a^	0.08 ± 0.01 ^a^	0.09 ± 0.02 ^a^	*

All values are mean ± standard deviation of triplicates. * There were no significant differences (*p* > 0.05) across samples with the same letter configuration in the same row; *p*-value: ** (*p* < 0.01), * (*p* < 0.05), ns (not significant).

**Table 5:** Physico-chemical characteristics of semi-smoked sausage produced with different amounts of PEG.

Parameters	Control	PEG5	PEG10	PEG15	PEG20	PEG25	*p* Value
WHC (%)	75.03 ± 0.71 ^a^	73.87 ± 0.33 ^b^	74.52 ± 0.34 ^a^	76.68 ± 0.24 ^c^	75.41 ± 0.21 ^a^	74.47 ± 0.74 ^a^	**
Emulsion Stability							
Fat exudation (%)	6.45 ± 0.74 ^c^	3.56 ± 0.98 ^a^	2.62 ± 0.77 ^b^	1.22 ± 0.12 ^a^	1.20 ± 1.11 ^a^	1.14 ± 1.01 ^a^	**
Water exudation (%)	20.45 ± 0.37 ^c^	15.31 ± 0.63 ^a^	9.33 ± 0.74 ^b^	6.40 ± 0.44 ^d^	7.22 ± 0.14 ^d^	7.25 ± 0.66 ^d^	**
CL (%)	11.12 ± 1.34 ^a^	9.45 ± 1.13 ^b^	6.53 ± 1.11 ^a^	4.96 ± 0.75 ^a^	5.73 ± 0.54 ^a^	5.45 ± 1.45 ^b^	*

All values are mean ± standard deviation of triplicates. * Different superscripts indicate statistically significant (*p* < 0.05) differences between means in the same row (a–d). *p*-value: ** (*p* < 0.01), * (*p* < 0.05), ns (not significant).

**Table 6:** Texture profile analysis of semi-smoked sausages.

Parameters	Control	PEG5	PEG10	PEG15	PEG20	PEG25	*p* Value
Hardness (N)	66.23 ± 1.65 ^b^	65.78 ± 1.47 ^b^	63.89 ± 1.05 ^a^	62.77 ± 1.08 ^a^	60.19 ± 1.38 ^c^	60.51 ± 1.05 ^b^	*
Springiness (mm)	0.90 ± 0.01 ^ab^	0.89 ± 0.05 ^b^	0.90 ± 0.02 ^b^	0.93 ± 0.03 ^a^	0.90 ± 0.01 ^b^	0.89 ± 0.01 ^a^	***
Cohesiveness	0.77 ± 0.01 ^b^	0.78 ± 0.00 ^b^	0.78 ± 0.01 ^ac^	0.83 ± 0.05 ^a^	0.81 ± 0.00 ^a^	0.82 ± 0.02 ^ac^	**
Gumminess (N)	22.45 ± 1.85 ^a^	23.72 ± 0.08 ^ac^	22.12 ± 0.07 ^c^	17.83 ± 0.14 ^c^	17.24 ± 0.12 ^a^	16.05 ± 0.02 ^c^	*
Chewiness (N × mm)	19.21 ± 2.59 ^a^	16.54 ± 0.15 ^a^	15.68 ± 0.16 ^a^	13.65 ± 0.21 ^ac^	13.20 ± 0.53 ^a^	12.98 ± 0.08 ^c^	*

All values are mean ± standard deviation of triplicates. * Different superscripts indicate statistically significant (*p* < 0.05) differences between means in the same row (a–c). *p*-value: *** (*p* < 0.001), ** (*p* < 0.01), * (*p* < 0.05), ns (not significant).

## Data Availability

The authors confirm that the data supporting the findings of this study are available within the article and its supplementary material.

## Supplementary Materials

The following supporting information can be downloaded at: https://www.mdpi.com/article/10.3390/foods11233886/s1, Table S1: Color parameters (*L**, *a**, *b**) and pH of pumpkin-based emulsion gel (PEG), and horse fat (HF) at 25 °C (solid-state).
