# Supplementary material for: A Pumpkin-Based Emulsion Gel as a Texture Improvement of Mixed Horsemeat Semi-Smoked Sausages
Source: Foods. 2022 Dec 1;11(23):3886. doi: 10.3390/foods11233886 (PMC9740514; doi:10.3390/foods11233886)
Supplement: Supplementary file 1 [file foods-11-03886-s001.zip › foods-2032954-supplementary.pdf]

Supplementary Material S1

Table S1. Color parameters (L*, a*, b*) and pH of pumpkin-based emulsion gel (PEG), and horse fat (HF) at 25 ⁰C (solid-state).

Parameters	PEG	HF
L*	66.66±0.59[a]	57.7±6.49[a]
a*	1.36±0.15[b]	-4.97±1.48[a]
b*	7.90±1.24[b]	-11.94±7.17[ab]
pH	6.37±0.25[a]	6.51±0.13[a]

Samples with identical letters in the same row did not present statistical differences ($p > 0.05$).
